# Supplementary material for: From sequence to enzyme mechanism using multi-label machine learning
Source: BMC Bioinformatics. 2014 May 19;15:150. doi: 10.1186/1471-2105-15-150 (PMC4229970; doi:10.1186/1471-2105-15-150)
Supplement: Additional file 2 — Java code of ml2db. Additional file ml2db_code.tar.gz contains the Java source code to run the multi-label machine learning experiments and save the results to database. The code’s Javadoc is included. [file 1471-2105-15-150-S2.zip › additional file 2/ml2db/ml2db/doc/test/ml2db/mulan/mulanarff/MulanArffTest.html]

MulanArffTest


JavaScript is disabled on your browser.


- Overview
- Package
- Class
- Use
- Tree
- Deprecated
- Index
- Help

- Prev Class
- Next Class

- Frames
- No Frames

- All Classes

- Summary:
- Nested |
- Field |
- Constr |
- Method

- Detail:
- Field |
- Constr |
- Method


test.ml2db.mulan.mulanarff

## Class MulanArffTest

- java.lang.Object
- - junit.framework.Assert
  - - junit.framework.TestCase
    - - test.ml2db.mulan.mulanarff.MulanArffTest

- All Implemented Interfaces:
  :   junit.framework.Test

  ---

    

  ```
  public class MulanArffTest
  extends junit.framework.TestCase
  ```

  Class

  Version:
  :   24 Feb 2011

  Author:
  :   Luna De Ferrari ldf(at)st-andrews.ac.uk

- - ### Field Summary

    Fields

    | Modifier and Type | Field and Description |
    | --- | --- |
    | `static java.lang.String` | `arff_EC_Instance1` |
    | `static java.lang.String` | `arffInstance1` |
    | `static java.lang.String` | `arffInstance2` |
    | `static java.lang.String` | `arffInstance4` |
    | `static java.lang.String` | `arffInstanceAttributeVoid` |
    | `static java.lang.String` | `arffInstanceClassAttributeVoid` |
    | `static java.lang.String` | `arffInstanceClassVoid` |
    | `static java.lang.String` | `ATT_EC_1_TO_STRING` |
    | `static java.lang.String` | `ATT_EC_11_TO_STRING` |
    | `static java.lang.String` | `ATT_EC_111_TO_STRING` |
    | `static java.lang.String` | `ATT_EC_1111_TO_STRING` |
    | `static java.lang.String` | `ATT_EC_2_TO_STRING` |
    | `static java.lang.String` | `ATT_EC_22_TO_STRING` |
    | `static java.lang.String` | `ATT_EC_222_TO_STRING` |
    | `static java.lang.String` | `ATT_EC_2222_TO_STRING` |
    | `static java.lang.String` | `ATT1_TO_STRING` |
    | `static java.lang.String` | `ATT2_TO_STRING` |
    | `static java.lang.String` | `ATT3_TO_STRING` |
    | `static java.lang.String` | `ATT4_TO_STRING` |
    | `static java.lang.String` | `ATT5_TO_STRING` |
  - ### Constructor Summary

    Constructors

    Constructor and Description

    `MulanArffTest()`
  - ### Method Summary

    Methods

    | Modifier and Type | Method and Description |
    | --- | --- |
    | `static int` | `generateTestArffAndReturnId(java.lang.String arffPropsPath)` Create test arff file |
    | `static java.lang.String` | `getExpectedMulanTestFile()` |
    | `static java.lang.String` | `getExpectedMulanTrainFile()` |
    | `static java.lang.String` | `getExpectedMulanXml2()` |
    | `static java.lang.String` | `getExpectedMulanXmlTestFile()` |
    | `static java.lang.String` | `getExpectedMulanXmlTrainFile()` |
    | `static int` | `getFirstTestArffFromDb()` public static int getFirstFilteredTestArff() { ArffPropsTableReader reader = ArffPropsTableReaderTest.getReader1(); // return the first arff having different identifiers as "old test id" // and "id the attributes were taken from (train set)" // return the first arff id containing "test" in its pathname return reader.getIdByArffPath("\_with\_attr\_of\_"); } public static int getFirstFilteredTrainArff() { ArffPropsTableReader reader = ArffPropsTableReaderTest.getReader1(); // return the first arff having different identifiers as "old test id" // and "id the attributes were taken from (train set)" // return the first arff id containing "test" in its pathname return reader.getIdByArffPath("\_with\_attr\_of\_"); } |
    | `static int` | `getFirstTrainArffFromDb()` |
    | `static int` | `getFirstValidArff2FromDb()` |
    | `static MulanArff` | `getLoadedMulanArff(java.lang.String arffPropsPath, MulanArff arff)` |
    | `static MulanArff` | `getMulanArff(java.lang.String arffPropsPath)` |
    | `static MulanArff` | `getMulanArff1()` |
    | `static MulanArff` | `getMulanArff1LoadedAndWritten()` |
    | `static MulanArff` | `getMulanArffNumericAttributes()` |
    | `static MulanArff` | `getMulanArffOldTestset()` |
    | `static MulanArff` | `getMulanArffTestsetLoadedAndWritten()` |
    | `static MulanArff` | `getMulanArffTrainLoadedAndWritten()` |
    | `static MulanArff` | `getMulanArffTrainset()` |
    | `static MulanArff` | `getWrittenMulan(MulanArff arff)` |
    | `void` | `testGenerateArffFileFromDb2()` public static String getExpectedMulanArff1() { return "@relation test\_data\n\n" + MulanArffTest.ATT1\_TO\_STRING + "\n" + MulanArffTest.ATT2\_TO\_STRING + "\n" + MulanArffTest.ATT3\_TO\_STRING + "\n" + MulanArffTest.ATT4\_TO\_STRING + "\n" + MulanArffTest.ATT5\_TO\_STRING + "\n" + "@attribute class1 {0,1}\n" + "@attribute class2 {0,1}\n" + "@attribute class3 {0,1}\n" + "@attribute class4 {0,1}\n" + "\n@data\n" + "% " + DataOne.INST1 + "\n" + MulanArffTest.arffInstance1 + "\n" + "% " + DataOne.INST2 + "\n" + MulanArffTest.arffInstance2 + "\n" + "% " + DataOne.INST4 + "\n" + MulanArffTest.arffInstance4 + "\n" + "% " + DataOne.INSTATTVOID + "\n" + MulanArffTest.arffInstanceAttributeVoid + "\n" + "% " + DataOne.INSTCLASSATTVOID + "\n" + MulanArffTest.arffInstanceClassAttributeVoid + "\n" + "% " + DataOne.INSTCLASSVOID + "\n" + MulanArffTest.arffInstanceClassVoid; } public static String getExpectedMulanArff2() { String attributes = "@relation test\_ec\_data\n\n@attribute 1.-.-.- {0,1}\n@attribute 1.1.-.- {0,1}\n@attribute 1.1.1.- {0,1}\n@attribute 1.1.1.1 {0,1}\n@attribute 2.-.-.- {0,1}\n@attribute 2.2.-.- {0,1}\n@attribute 2.2.2.- {0,1}\n@attribute 2.2.2.2 {0,1}\n@attribute 3.-.-.- {0,1}\n@attribute 3.3.-.- {0,1}\n@attribute 3.3.3.- {0,1}\n@attribute 3.3.3.3 {0,1}\n@attribute att1 {0,1}\n@attribute att2 {0,1}\n@attribute att3 {0,1}\n@attribute att4 {0,1}\n@attribute att5 {0,1}\n\n" ; String instances = "@data\n% inst1\n{0 1,1 1,2 1,3 1,12 1}\n% inst10\n{4 1,5 1,6 1,7 1,16 1}\n% inst11\n{4 1,5 1,6 1,7 1}\n% inst12\n{12 1}\n% inst13\n{}\n% inst2\n{4 1,5 1,6 1,7 1,14 1}\n% inst3\n{0 1,1 1,2 1,3 1,12 1}\n% inst4\n{4 1,5 1,6 1,7 1,14 1}\n% inst5\n{0 1,1 1,2 1,3 1,12 1}\n% inst6\n{4 1,5 1,6 1,7 1,14 1}\n% inst7\n{0 1,1 1,2 1,3 1,12 1}\n% inst8\n{4 1,5 1,6 1,7 1,14 1}\n% inst9\n{0 1,1 1,2 1,3 1,15 1}\n% instA\n{8 1,9 1,10 1,11 1,13 1}\n% instB\n{4 1,5 1,6 1,7 1,14 1}\n% instC\n{4 1,5 1,6 1,7 1,13 1,14 1}\n% inst\_att\_void\n{4 1,5 1,6 1,7 1}\n% inst\_class\_att\_void\n{}\n% inst\_class\_void\n{13 1}" ; return attributes + instances; } |
    | `void` | `testGenerateMulanArffNumericAttributes()` |

    - ### Methods inherited from class junit.framework.TestCase

      `countTestCases, getName, run, run, runBare, setName, toString`
    - ### Methods inherited from class junit.framework.Assert

      `assertEquals, assertEquals, assertEquals, assertEquals, assertEquals, assertEquals, assertEquals, assertEquals, assertEquals, assertEquals, assertEquals, assertEquals, assertEquals, assertEquals, assertEquals, assertEquals, assertEquals, assertEquals, assertEquals, assertEquals, assertFalse, assertFalse, assertNotNull, assertNotNull, assertNotSame, assertNotSame, assertNull, assertNull, assertSame, assertSame, assertTrue, assertTrue, fail, fail, failNotEquals, failNotSame, failSame, format`
    - ### Methods inherited from class java.lang.Object

      `equals, getClass, hashCode, notify, notifyAll, wait, wait, wait`

- - ### Field Detail


    - #### arffInstance1

      ```
      public static final java.lang.String arffInstance1
      ```

      See Also:
      :   Constant Field Values


    - #### arff\_EC\_Instance1

      ```
      public static final java.lang.String arff_EC_Instance1
      ```

      See Also:
      :   Constant Field Values


    - #### arffInstance2

      ```
      public static final java.lang.String arffInstance2
      ```

      See Also:
      :   Constant Field Values


    - #### arffInstance4

      ```
      public static final java.lang.String arffInstance4
      ```

      See Also:
      :   Constant Field Values


    - #### arffInstanceClassAttributeVoid

      ```
      public static final java.lang.String arffInstanceClassAttributeVoid
      ```

      See Also:
      :   Constant Field Values


    - #### arffInstanceClassVoid

      ```
      public static final java.lang.String arffInstanceClassVoid
      ```

      See Also:
      :   Constant Field Values


    - #### arffInstanceAttributeVoid

      ```
      public static final java.lang.String arffInstanceAttributeVoid
      ```

      See Also:
      :   Constant Field Values


    - #### ATT1\_TO\_STRING

      ```
      public static final java.lang.String ATT1_TO_STRING
      ```

      See Also:
      :   Constant Field Values


    - #### ATT2\_TO\_STRING

      ```
      public static final java.lang.String ATT2_TO_STRING
      ```

      See Also:
      :   Constant Field Values


    - #### ATT3\_TO\_STRING

      ```
      public static final java.lang.String ATT3_TO_STRING
      ```

      See Also:
      :   Constant Field Values


    - #### ATT4\_TO\_STRING

      ```
      public static final java.lang.String ATT4_TO_STRING
      ```

      See Also:
      :   Constant Field Values


    - #### ATT5\_TO\_STRING

      ```
      public static final java.lang.String ATT5_TO_STRING
      ```

      See Also:
      :   Constant Field Values


    - #### ATT\_EC\_1\_TO\_STRING

      ```
      public static final java.lang.String ATT_EC_1_TO_STRING
      ```

      See Also:
      :   Constant Field Values


    - #### ATT\_EC\_11\_TO\_STRING

      ```
      public static final java.lang.String ATT_EC_11_TO_STRING
      ```

      See Also:
      :   Constant Field Values


    - #### ATT\_EC\_111\_TO\_STRING

      ```
      public static final java.lang.String ATT_EC_111_TO_STRING
      ```

      See Also:
      :   Constant Field Values


    - #### ATT\_EC\_1111\_TO\_STRING

      ```
      public static final java.lang.String ATT_EC_1111_TO_STRING
      ```

      See Also:
      :   Constant Field Values


    - #### ATT\_EC\_2\_TO\_STRING

      ```
      public static final java.lang.String ATT_EC_2_TO_STRING
      ```

      See Also:
      :   Constant Field Values


    - #### ATT\_EC\_22\_TO\_STRING

      ```
      public static final java.lang.String ATT_EC_22_TO_STRING
      ```

      See Also:
      :   Constant Field Values


    - #### ATT\_EC\_222\_TO\_STRING

      ```
      public static final java.lang.String ATT_EC_222_TO_STRING
      ```

      See Also:
      :   Constant Field Values


    - #### ATT\_EC\_2222\_TO\_STRING

      ```
      public static final java.lang.String ATT_EC_2222_TO_STRING
      ```

      See Also:
      :   Constant Field Values
  - ### Constructor Detail


    - #### MulanArffTest

      ```
      public MulanArffTest()
      ```
  - ### Method Detail


    - #### testGenerateArffFileFromDb2

      ```
      public void testGenerateArffFileFromDb2()
      ```

      public static String getExpectedMulanArff1() { return
      "@relation test\_data\n\n" + MulanArffTest.ATT1\_TO\_STRING + "\n" +
      MulanArffTest.ATT2\_TO\_STRING + "\n" + MulanArffTest.ATT3\_TO\_STRING + "\n"
      + MulanArffTest.ATT4\_TO\_STRING + "\n" + MulanArffTest.ATT5\_TO\_STRING +
      "\n" + "@attribute class1 {0,1}\n" + "@attribute class2 {0,1}\n" +
      "@attribute class3 {0,1}\n" + "@attribute class4 {0,1}\n" + "\n@data\n" +
      "% " + DataOne.INST1 + "\n" + MulanArffTest.arffInstance1 + "\n" + "% " +
      DataOne.INST2 + "\n" + MulanArffTest.arffInstance2 + "\n" + "% " +
      DataOne.INST4 + "\n" + MulanArffTest.arffInstance4 + "\n" + "% " +
      DataOne.INSTATTVOID + "\n" + MulanArffTest.arffInstanceAttributeVoid +
      "\n" + "% " + DataOne.INSTCLASSATTVOID + "\n" +
      MulanArffTest.arffInstanceClassAttributeVoid + "\n" + "% " +
      DataOne.INSTCLASSVOID + "\n" + MulanArffTest.arffInstanceClassVoid; }
      public static String getExpectedMulanArff2() { String attributes =
      "@relation test\_ec\_data\n\n@attribute 1.-.-.- {0,1}\n@attribute 1.1.-.- {0,1}\n@attribute 1.1.1.- {0,1}\n@attribute 1.1.1.1 {0,1}\n@attribute 2.-.-.- {0,1}\n@attribute 2.2.-.- {0,1}\n@attribute 2.2.2.- {0,1}\n@attribute 2.2.2.2 {0,1}\n@attribute 3.-.-.- {0,1}\n@attribute 3.3.-.- {0,1}\n@attribute 3.3.3.- {0,1}\n@attribute 3.3.3.3 {0,1}\n@attribute att1 {0,1}\n@attribute att2 {0,1}\n@attribute att3 {0,1}\n@attribute att4 {0,1}\n@attribute att5 {0,1}\n\n"
      ; String instances =
      "@data\n% inst1\n{0 1,1 1,2 1,3 1,12 1}\n% inst10\n{4 1,5 1,6 1,7 1,16 1}\n% inst11\n{4 1,5 1,6 1,7 1}\n% inst12\n{12 1}\n% inst13\n{}\n% inst2\n{4 1,5 1,6 1,7 1,14 1}\n% inst3\n{0 1,1 1,2 1,3 1,12 1}\n% inst4\n{4 1,5 1,6 1,7 1,14 1}\n% inst5\n{0 1,1 1,2 1,3 1,12 1}\n% inst6\n{4 1,5 1,6 1,7 1,14 1}\n% inst7\n{0 1,1 1,2 1,3 1,12 1}\n% inst8\n{4 1,5 1,6 1,7 1,14 1}\n% inst9\n{0 1,1 1,2 1,3 1,15 1}\n% instA\n{8 1,9 1,10 1,11 1,13 1}\n% instB\n{4 1,5 1,6 1,7 1,14 1}\n% instC\n{4 1,5 1,6 1,7 1,13 1,14 1}\n% inst\_att\_void\n{4 1,5 1,6 1,7 1}\n% inst\_class\_att\_void\n{}\n% inst\_class\_void\n{13 1}"
      ;
      return attributes + instances; }


    - #### testGenerateMulanArffNumericAttributes

      ```
      public void testGenerateMulanArffNumericAttributes()
      ```


    - #### generateTestArffAndReturnId

      ```
      public static int generateTestArffAndReturnId(java.lang.String arffPropsPath)
      ```

      Create test arff file

      Returns:
      :   the identifier of the arff file record in the database


    - #### getExpectedMulanTestFile

      ```
      public static java.lang.String getExpectedMulanTestFile()
      ```


    - #### getExpectedMulanTrainFile

      ```
      public static java.lang.String getExpectedMulanTrainFile()
      ```


    - #### getExpectedMulanXml2

      ```
      public static java.lang.String getExpectedMulanXml2()
      ```


    - #### getExpectedMulanXmlTestFile

      ```
      public static java.lang.String getExpectedMulanXmlTestFile()
      ```


    - #### getExpectedMulanXmlTrainFile

      ```
      public static java.lang.String getExpectedMulanXmlTrainFile()
      ```


    - #### getFirstTestArffFromDb

      ```
      public static int getFirstTestArffFromDb()
      ```

      public static int getFirstFilteredTestArff() { ArffPropsTableReader
      reader = ArffPropsTableReaderTest.getReader1(); // return the first arff
      having different identifiers as "old test id" // and
      "id the attributes were taken from (train set)"
      // return the first arff id containing "test" in its pathname return
      reader.getIdByArffPath("\_with\_attr\_of\_"); }
      public static int getFirstFilteredTrainArff() { ArffPropsTableReader
      reader = ArffPropsTableReaderTest.getReader1(); // return the first arff
      having different identifiers as "old test id" // and
      "id the attributes were taken from (train set)"
      // return the first arff id containing "test" in its pathname return
      reader.getIdByArffPath("\_with\_attr\_of\_"); }


    - #### getFirstTrainArffFromDb

      ```
      public static int getFirstTrainArffFromDb()
      ```


    - #### getFirstValidArff2FromDb

      ```
      public static int getFirstValidArff2FromDb()
      ```


    - #### getLoadedMulanArff

      ```
      public static MulanArff getLoadedMulanArff(java.lang.String arffPropsPath,
                                 MulanArff arff)
      ```


    - #### getMulanArff

      ```
      public static MulanArff getMulanArff(java.lang.String arffPropsPath)
      ```


    - #### getMulanArff1

      ```
      public static MulanArff getMulanArff1()
      ```


    - #### getMulanArff1LoadedAndWritten

      ```
      public static MulanArff getMulanArff1LoadedAndWritten()
      ```


    - #### getMulanArffNumericAttributes

      ```
      public static MulanArff getMulanArffNumericAttributes()
      ```


    - #### getMulanArffOldTestset

      ```
      public static MulanArff getMulanArffOldTestset()
      ```


    - #### getMulanArffTestsetLoadedAndWritten

      ```
      public static MulanArff getMulanArffTestsetLoadedAndWritten()
      ```


    - #### getMulanArffTrainLoadedAndWritten

      ```
      public static MulanArff getMulanArffTrainLoadedAndWritten()
      ```


    - #### getMulanArffTrainset

      ```
      public static MulanArff getMulanArffTrainset()
      ```


    - #### getWrittenMulan

      ```
      public static MulanArff getWrittenMulan(MulanArff arff)
      ```


- Overview
- Package
- Class
- Use
- Tree
- Deprecated
- Index
- Help

- Prev Class
- Next Class

- Frames
- No Frames

- All Classes

- Summary:
- Nested |
- Field |
- Constr |
- Method

- Detail:
- Field |
- Constr |
- Method
